# Nailfold Video-Capillaroscopy in Sarcoidosis: New Perspectives and Challenges

**DOI:** 10.3390/tomography10100114

**Published:** 2024-09-25

**Authors:** Maria Chianese, Gianluca Screm, Paola Confalonieri, Francesco Salton, Liliana Trotta, Beatrice Da Re, Antonio Romallo, Alessandra Galantino, Mario D’Oria, Michael Hughes, Giulia Bandini, Marco Confalonieri, Elisa Baratella, Lucrezia Mondini, Barbara Ruaro

**Affiliations:** 1 Pulmonology Unit, Department of Medical Surgical and Health Sciences, University of Trieste, 34149 Trieste, Italy; 2 Division of Vascular and Endovascular Surgery, University Hospital of Cattinara, 34149 Trieste, Italy; 3 Division of Musculoskeletal and Dermatological Sciences, Faculty of Biology, Medicine and Health, The University of Manchester & Salford Royal NHS Foundation Trust, Manchester M6 8HD, UK; 4 Department of Experimental and Clinical Medicine, Division of Internal Medicine, Azienda Ospedaliero Universitaria Careggi and University of Florence, 50134 Florence, Italy; 5 Radiology Unit, Department of Medical Surgical and Health Sciences, University Hospital of Cattinara, 34149 Trieste, Italy

**Keywords:** sarcoidosis, nailfold video-capillaroscopy (NVC), connective tissue disease, inflammatory disease

## Abstract

Introduction: Nailfold video-capillaroscopy (NVC) is a non-invasive cost-effective technique involving the microscopic examination of small blood vessels of the distal nailfold with a magnification device. It provides valuable information regarding the microcirculation including anomalies such as tortuous or dilated capillaries, hemorrhages, and avascular areas, which can characterize connective tissue diseases. The utility of NVC in the diagnosis and monitoring of systemic sclerosis (SSc) has been investigated in numerous studies allowing the distinction of the specific microvascular pattern of scleroderma from different conditions other than scleroderma (non-scleroderma pattern). Sarcoidosis (SA) is a systemic inflammatory disease that can affect various organs, including the lungs, skin, and lymph nodes. The purpose of our review was to evaluate the current state of the art in the use of NVC in the diagnosis of SA, to understand the indications for its use and any consequent advantages in the management of the disease in different settings in terms of benefits for patients. Materials and Methods: We searched for the key terms “sarcoidosis” and “video-capillaroscopy” in a computerized search of Pub-Med, extending the search back in time without setting limits. We provided a critical overview of the literature, based on a precise evaluation. After our analysis, we examined the six yielded works looking for answers to our questions. Results: Few studies have evaluated that microcirculation is often compromised in SA, with alterations in blood flow and consequent tissue damage. Discussion: Basing on highlighted findings, NVC appears to be a useful tool in the initial evaluation of sarcoidosis patients. Furthermore, capillaroscopy is useful in the evaluation of the coexistence of sarcoidosis and scleroderma spectrum disorder or overlap syndromes. Conclusions: In conclusions, no specific pattern has been described for sarcoidosis, and further re-search is needed to fully understand the implications of nailfold capillaroscopy find-ings in this disease and to establish standardized guidelines for its use in clinical practice.

## 1. Introduction

Nailfold capillaroscopy (NVC) is a non-invasive cost-effective technique involving the microscopic examination of small blood vessels of the distal nailfold with a magnification device. It provides valuable information regarding the microcirculation including anomalies such as tortuous or dilated capillaries, hemorrhages, and avascular areas, which can characterize connective tissue diseases [1,2,3].

Sarcoidosis (SA) is a systemic inflammatory disease that can affect various organs, including the lungs, skin, and lymph nodes [4,5,6,7,8,9,10].

The microcirculation is often compromised in SA, with alterations in blood flow and consequent tissue damage. Several underlying mechanisms include obstruction by granulomas, vasculitis, and endothelial dysfunction [11,12,13,14,15,16,17,18,19,20,21].

The purpose of our review was to evaluate the current state of the art in the use of NVC in the diagnosis and follow-up of SA, to understand the indications for its use and any consequent advantages in the management of the disease in different settings in terms of benefits for patients and savings in time and money.

### 1.1. Nailfold Video-Capillaroscopy the History of Invention and Development of the Technique

The utility of NVC in the diagnosis and monitoring of systemic sclerosis (SSc) has been investigated in numerous studies allowing the distinction of the specific microvascular pattern of scleroderma from different conditions other than scleroderma (non-scleroderma pattern). In fact, capillaroscopy has been recognized among the classification criteria for systemic sclerosis since 2013 (American College of Rheumatology (ACR)/European League Against Rheumatism (EULAR) criteria for the classification of systemic sclerosis) [22]. In addition, current evidence includes NVC in the management of scleroderma patients [23,24,25,26]. However, only little information is available about NVC in SA.

Nailfold video-capillaroscopy (NVC) is a non-invasive imaging technique that allows the examination of capillaries located at the nailfold, which is the area of skin at the base of the nail. This technique has evolved significantly since its inception, and it has become an essential tool in both clinical and research settings for assessing microcirculation and diagnosing various vascular and autoimmune diseases.

The origins of capillaroscopy can be traced back to the early 20th century when researchers began recognizing the importance of small blood vessels (capillaries) in microcirculation. The first attempts to visualize these tiny vessels were limited by the optical technology of the time. In the 1950s, traditional capillaroscopy was used, often involving the use of a simple light microscope with a magnifying lens to examine the capillaries under the nailfold. This method enabled basic observation but had significant limitations in resolution and quantification. As optical and imaging technologies improved, the field of capillaroscopy advanced. The introduction of more sophisticated microscopes equipped with photographic capabilities and better light sources led to increased interest and more detailed studies of capillary morphology and function [1,2].

**Figure 1:**
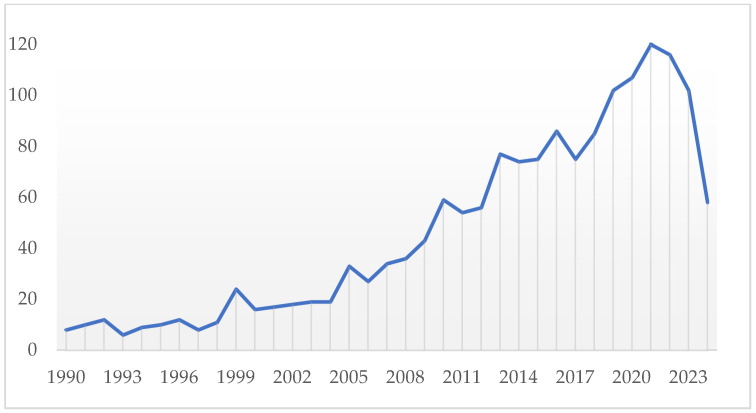
Publication trend concerning NVC over the years, from 1990 to the present day, which shows the growing interest in this technique. The x-axis shows the years from 1990 to the present, the y-axis the number of publications according to PubMed, Scopus and Google Scholar.

The specific technique of nailfold video-capillaroscopy emerged in the late 20th century, particularly in the 1990s, when video technology became more widespread, Figure 1 shows the publication trend concerning NVC over the years, from 1990 to the present day, which shows the growing interest in this technique (Figure 1). The incorporation of digital cameras allowed for higher resolution imaging, real-time observation, and the ability to record and analyze capillary structure and function effectively [1,2].

Nailfold video-capillaroscopes typically provide a magnification of 50× to 500×, enabling detailed views of capillary networks. The resolution achieved allows for the identification of capillary density, size, shape, and branching patterns. Modern NVC systems incorporate high-resolution digital cameras and software that can capture images and videos in real time. These images can be analyzed using specialized software, which quantifies parameters such as the number of capillaries per unit area, the diameter of capillaries, and blood flow characteristics [1,2]. Over the years, efforts have been made to standardize the technique in terms of patient positioning, lighting, and analysis methods, which has enhanced the reproducibility of results across different studies and clinical settings. NVC is a critical tool for diagnosing and monitoring various conditions, particularly microvascular diseases like systemic sclerosis, dermatomyositis, and Raynaud’s phenomenon. The findings from NVC can indicate early signs of vascular involvement and help in risk stratification. In addition to its diagnostic utility, NVC is employed in clinical research to study the microvascular changes associated with various diseases, environmental factors, and therapeutic interventions. Researchers can correlate capillary morphology with systemic diseases and inflammatory processes [1,2]. NVC can be used to evaluate the effectiveness of therapies targeting vascular function or inflammation. Changes in capillary structure or density can be followed over time, providing insights into the effectiveness of treatments. The development of nailfold video-capillaroscopy continues to evolve with the integration of advanced imaging technologies, such as higher resolution cameras and software that utilize artificial intelligence for pattern recognition and automated analysis. Continuous efforts aim to establish NVC as a more widespread tool in routine clinical practice, potentially allowing for early diagnosis of microvascular involvement and enhancing personalized therapy for patients. In conclusion, nailfold video-capillaroscopy has established itself as a valuable technique in both clinical and research settings, providing insights into the microvascular landscape that are crucial for understanding a wide range of health conditions. Its history reflects the broader trends in medical imaging and technology, leading to a sophisticated approach that enhances diagnostic accuracy and therapeutic monitoring in modern medicine [1,2]. Recently, numerous methods were described to perform nailfold capillaroscopy including the stereomicroscope, dermatoscope, ophthalmoscope, smartphone devices, digital USB microscopes, and the current gold-standard device, the nailfold video-capillaroscope [27].

### 1.2. The Capillaroscopy Applications

Video-capillaroscopy is carried out with a handheld device which can be moved along the nailfold, which also allows images to be taken of systemic sclerosis patients with severe flexion contractures and can be learnt fast [28,29]. It combines a microscope and a digital video camera with a magnification between 50× and 500×. The acquired images are stored and analyzed on specific parameters in each single frame separately, and the software can combine adjacent images to visualize the entire nailfold [30].

Morphological evaluation of skin capillaries in Raynaud’s phenomenon is generally performed at the nailfold because the fingers are involved in the pathological process of some autoimmune disorders which give rise to secondary Raynaud’s phenomenon; they are a segment of the body easy to analyze by video-capillaroscopy and because it is possible to define accurately the observation site [31].

Before starting the video-capillaroscopy, the patient is acclimatized in a room with a constant temperature of 20–22 °C for at least 15 min so that undue vasoconstriction of capillaries, which can induce false positivity for avascular zones, can be avoided [32]. Few studies exist on changes in blood flow secondary to changes in body temperature, which have only been partially investigated with capillaroscopy. The capillaroscopy in standard conditions cannot measure the flow because it does not have the device that quantifies the velocity of the blood over time. The standard NVC tool allows only a descriptive evaluation of the flow (e.g., slowed, granular, etc.) to be given. Furthermore, flow assessment is not standardized by current EULAR guidelines [1,2,3,29,30,31,32,33,34]. Nevertheless, there are other promising methods for visualizing microcirculation, including the hybrid photoacoustic ultrasound imaging system [35]. The epidermis is normally transparent, and application of oil smoothens the surface permitting the observation of superficial structures of the skin.

**Figure 2:**
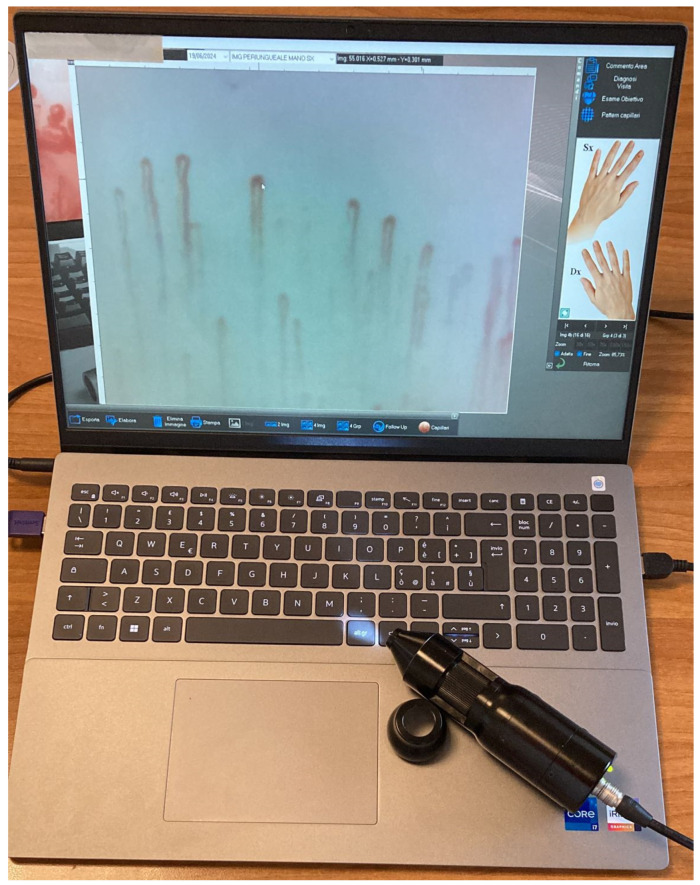
The figure shows the components for performing a capillaroscopy: a video-capillaroscopic probe with a magnifying lens (×50 to ×500) is used, equipped with a focusing ring and light adjustment system. The probe is connected to a screen to display real-time images and capture them via a foot pedal. The images are then analyzed using image digitizing software (Software Videocap 200® Reumatologia Release 11.00.02), which allows measurements and capillary counts to be taken.

The nailfold video-capillaroscope comprises several components: a high-resolution video-capillaroscope with lenses ranging from ×50 to ×500, integrated into the probe; a 12-LED light powered by a USB cable, with a knob to adjust light intensity; a high-resolution color screen for image observation; a Deskjet printer with photographic quality; digital image analysis software; and a footswitch for capturing and storing images when the desired quality is achieved, as shown in Figure 2. Four images are taken per finger, allowing for the evaluation of all fingers.

The contact probe facilitates exploration of the entire nailbed, even in challenging cases such as flexion contractures. Image quality can be maximized by manually adjusting the probe’s focus, which can be monitored directly on the screen. Most experts agree that the best capillary views are obtained from the fourth finger [33].

Fingers are examined from left to right, from the operator’s point of view. According to some schools, the thumbs are not evaluated because capillaries are less well visualized there and usually, the 2nd, 3rd, 4th, and 5th fingers are examined resulting in 32 images which are stored.

**Figure 3:**
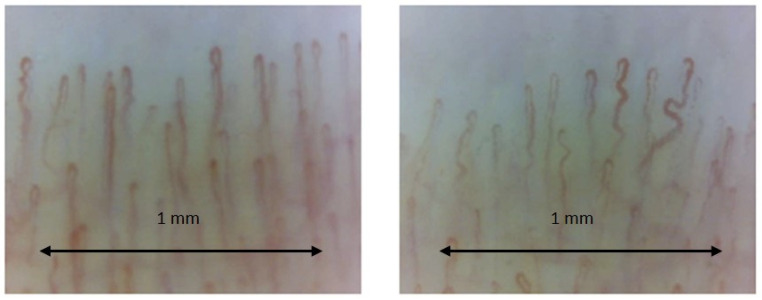
Normal capillary bed image showing good visibility of capillaries with regular architecture. The capillary count per linear mm results in 8–9 capillaries/mm (the number of capillaries is normal). Next to normal capillaries, there are some tortuous and crossed capillaries (magnification ×200). Operators: B.R. and L.M., Pulmonology Unit, University of Trieste).

The nailfold capillaries in healthy individuals typically exhibit an open hairpin shape. These capillaries are uniformly sized, regularly arranged in parallel lines, and generally number between 6 and 14. However, there is considerable variation within the normal range of morphology [34,36]. Normal nailfold bed is schematized in Table 1 and Figure 3.

Morphological anomalies are likewise detectable in healthy people, including hemorrhages and avascular areas, but certain other features, such as giant capillaries or extensive avascularity, do not seem to be present in the normal population and should be considered pathological [36].

Raynaud’s phenomenon is a clinical indicator of altered vascular tone control in the microcirculation. This condition may also manifest through telangiectasia on the face and hands, as well as the formation of digital pitting scars and ulcers. Initially, blood flow is reduced only during vasoconstriction due to functional alterations. Over time, pathological changes dramatically decrease microvessel patency and blood supply. Vascular smooth muscle cell proliferation leads to intimal growth, matrix deposition in the vessel wall, and eventual complete occlusion of the lumen. Perivascular inflammation may be linked to early endothelial damage, characterized by an exaggerated angiogenic response that later shifts to defective wound healing and fibrosis. In the microcirculation, this progression is marked by giant capillaries, microhemorrhages, architectural changes in the capillaries, and the loss of capillaries, known as desertification [37,38,39,40,41,42,43,44,45].

Raynaud’s phenomenon has a prevalence ranging between 3–22% in population-based surveys [38]. It is defined primary when it is benign, not related to any other disease, while it is secondary when related to conditions such as arterial or neurological disorders of the upper extremities, exposure to injurious occupational factors (vibration, microtrauma, and vinyl chloride), drug side effects (beta-blockers), oncological and hematological disorders, myeloproliferative disorders, paraneoplastic syndromes, and hypothyroidism [39]. Generally, it is a manifestation of an underlying collagen vascular disease and frequently precedes the underlying disease by many years [31].

Most patients with clinically recognizable systemic sclerosis exhibit distinctive combinations of nailfold capillary abnormalities that are easily evaluated through pattern recognition. Maricq et al. described the scleroderma pattern using the wide-field technique (magnification ×12–14). This pattern is pathognomonic and includes notable widening of all three segments of the capillary loop (arterial, venous, and intermediate), loss of capillaries, and disorganization of the nailfold capillary bed. Additionally, many branched, “bushy” capillaries may also be observed [33]. Cutolo et al. introduced a system for the semi-quantitative evaluation of key parameters of the scleroderma pattern. This evaluation involves the measurement of the total number of capillaries, giant capillaries, microhemorrhages, and branching, and the scoring of a score ranging from 0 to 3. The score depends on the number of abnormalities present; in particular a score of 0 predicts the absence of abnormalities, a score of 1 is awarded if abnormalities are present in less than 33%, a score of 2 between 33% and 66%, and above 66% a score of 3 is awarded [1].

Scleroderma-type changes are also seen in diseases other than clinically recognizable systemic sclerosis, such as Raynaud’s phenomenon without a definite diagnosis of an associated disease, dermatomyositis, mixed CTD, and undifferentiated CTD, suggesting the sharing of common pathogenic factors and the belonging to the family of scleroderma spectrum (SDS) disorders [46,47,48,49,50,51,52,53,54,55,56,57,58,59,60,61].

**Figure 4:**
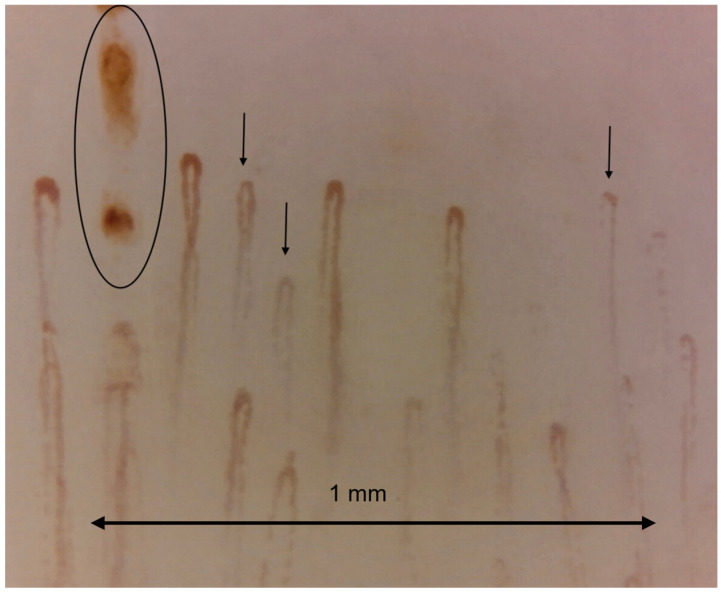
Capillaroscopic image at x200 showing granular blood flow, sign of slowing flow (marked with black arrow). microhemorrhage is also visible in the circle. Operators: B.R. and L.M., Pulmonology Unit, University of Trieste).

In contrast to SDS disorders, other connective tissue diseases (CTD) like systemic lupus erythematosus (SLE) and Sjögren’s syndrome do not have unique capillary patterns. Various capillary abnormalities have been observed in these conditions [48,49,50,51,52,53,54,55,56,57,58]. These abnormalities, on their own, are not predictive of any specific condition and are often referred to as non-specific. Generally, isolated or uncommon anomalies represent a variation of normal. However, when anomalies are numerous or when multiple anomalies occur in one individual, they indicate an underlying CTD [36]. In addition to systemic sclerosis, capillaroscopy has proved useful in a number of other diseases, especially in the context of rheumatic and vascular diseases, including dermatomyositis and polymyositis, rheumatoid arthritis and systemic lupus erythematosus, and antiphospholipid antibody syndrome. Capillaroscopy has an increasing role in microvascular analysis in diabetes mellitus, both type 1 and type 2. The main contributions of capillaroscopy in the study of diabetes concern the assessment of microvascular changes, the early detection of microcirculatory dysfunction, and the monitoring of disease progression. Also, with capillaroscopy, using a magnification of at least ×200, it is possible to visualize the aggregation of erythrocytes and capillaries or venules where blood flow is slowed or absent, and granular flow and plasma lacunae can also be visualized, as shown in Figure 4.

This method is useful for detecting microvascular alterations at an early stage and monitoring the progression of vascular damage, providing additional information for the clinical management of the diabetic patient. However, it is considered as a complementary method to other diagnostic techniques, including e.g., portable optical diffuse speckle pulsatile flowmetry [62]. Capillaroscopy has also proven to be a useful tool in adding information about arterial hypertension and microcirculation involvement, SARS-CoV-2 disease and some pulmonary diseases including COPD, pulmonary fibrosis, and pulmonary hypertension [63,64,65].

Characterized by non-invasiveness and cost-effectiveness and providing important information about disease activity and treatment response, NVC should be a useful tool also for evaluating the microcirculation in patients with SA; by providing insights into microvascular changes, capillaroscopy may help in the early detection of disease complications, assessing disease activity and severity, and monitoring treatment response.

### 1.3. NVC and the Other Imaging Systems, Like Photoacoustic Microscopy (PAM), Diffuse Speckle Pulsatile Flowmetry (DSPF) and Optical Coherence Tomography (OCT)

Capillaroscopy is an in vivo technique used to assess the microcirculation, primarily for the differential diagnosis of Raynaud’s phenomenon. Raynaud’s phenomenon is marked by digital blanching and cyanosis following cold exposure of the fingers or toes, resulting from vasospasm of the digital arteries [1,2,3]. This is followed by dilation of capillaries and venules during the ischemic phase. Upon re-warming, reactive hyperemia may occur, characterized by increased blood flow into the now-dilated arterioles and capillaries [59,60,61]. Other related imaging technologies are the following:

a- Photoacoustic Microscopy (PAM): combines optical and ultrasound imaging. It uses laser-induced ultrasound waves generated by the absorption of light, which can provide high-resolution images of tissue structures. This technique is particularly useful for imaging vascular structures, oxygenation levels, and tissue composition in real-time. It has applications in cancer research, dermatology, and other fields where vascular assessments are crucial [59,60,61].
b- Diffuse Speckle Pulsatile Flowmetry (DSPF): employs laser speckle contrast imaging to assess blood flow dynamics. It detects the movement of scatterers (like red blood cells) within a tissue, evaluating blood flow based on the temporal changes in speckle patterns. It is used to monitor microcirculation and perfusion in various tissues, which can be vital in wound healing, tissue engineering, and assessing vascular health.
c- Optical Coherence Tomography (OCT): OCT is an imaging technique that utilizes light waves to take cross-section images of biological tissues. It offers high-resolution images (in the micrometer range) without the need for contrast agents. Commonly used in ophthalmology for retinal imaging, OCT is being explored for skin imaging, cardiovascular assessments, and monitoring microvascular conditions similar to nailfold capillaroscopy.

These techniques present several different characteristics. In fact, while nailfold capillaroscopy provides good resolution for capillary architecture, PAM and OCT offer higher spatial resolution and depth information, useful for imaging larger and deeper structures. Techniques like PAM and DSPF can measure hemodynamic parameters in real-time, adding functional information about blood flow that nailfold capillaroscopy does not typically offer. Nailfold capillaroscopy is easier to perform and can be more accessible in clinical settings for direct examinations. In contrast, PAM, DSPF, and OCT may require more specialized equipment and training.

Integrating these imaging modalities can provide comprehensive insights into microvascular health, combining structural details from nailfold capillaroscopy with functional assessments from DSPF and PAM. OCT can add another layer of detail regarding tissue organization and pathology. This approach can enhance diagnostic accuracy, monitor disease progression, and evaluate treatment responses in conditions related to microvascular dysfunction.

In summary, while nailfold capillaroscopy plays a crucial role in assessing microvascular status, related technologies like PAM, DSPF, and OCT expand the horizons of vascular imaging, offering both structural and functional insights that can complement clinical evaluations.

### 1.4. The Sarcoidosis Disease

A significant difference in the epidemiology of sarcoidosis (SA) disease has been estimated across different countries worldwide, with greater rarity in East Asia, where the incidence does not exceed 1.3 cases per 100,000 people per year, and a recorded prevalence in Taiwan of 2.2 cases per 100,000 people. In contrast, the disease is more frequent in Scandinavian countries, where the incidence reaches 11.5 cases per 100,000 people per year and the prevalence in Sweden is 160 cases per 100,000 people [6]. This disease is reported as being more significantly associated with the female sex [7,8].

The onset typically occurs between the third and fifth decades of life; approximately 90 to 95% of patients show the involvement of the lung or thoracic lymph node usually presenting dyspnea, cough, fatigue, and chest pain which are often accompanied by systemic symptoms such as malaise, fever, fatigue, and weight loss [9,10,11,12].

Extrapulmonary manifestations at the onset of the disease are identifiable in about half of the cases in association with pulmonary symptoms, and in some cases, they are the sole initial manifestation. Skin lesions are the most frequent extrapulmonary manifestations, along with peripheral lymphadenomegaly and ocular alterations [10]. Mild hypercalcemia with hypercalciuria, swelling of the salivary glands, and facial nerve paralysis are also common. Some extrapulmonary manifestations are considered pathognomonic, such as Löfgren syndrome (5 to 10 percent of patients), uveoparotid fever, and lupus pernio [9,10,13,14,15].

In about half of the cases, sarcoidosis is incidentally discovered through chest imaging, which generally shows early radiological stages in patients with a normal pulmonary physical examination [9].

Upon targeted medical history, these patients may reveal previously overlooked symptoms, such as systemic symptoms or exertional dyspnea, skin rashes, peripheral lymphadenopathy, nephrolithiasis, uveitis, palpitations, and syncope [14].

Diagnosing sarcoidosis involves identifying compatible clinical and radiographic manifestations, ruling out other diseases with similar presentations, and typically detecting non-necrotizing granulomas through histopathology. A diagnostic score for sarcoidosis has been developed to help determine if multiple manifestations across different systems are specific enough to avoid further investigation [4,16].

With significant insights, the complex interplay of genetic, environmental, and immunological factors contributing to the pathogenesis of sarcoidosis has been proven in recent research. Genetic predisposition is crucial; variants in the human leukocyte antigen (HLA) genes are strongly associated with susceptibility and clinical manifestations of the disease. Next-generation sequencing has identified specific HLA polymorphisms linked to different phenotypes and disease outcomes; environmental factors, such as microbial agents and inorganic particles, are believed to trigger macrophages and T cells in genetically predisposed individuals, leading to the formation of granulomas. In fact, activated macrophages present antigens to CD4^+^ T cells, leading to the release of cytokines (IFN-γ, TNF-α) and interleukins (IL-2, IL-12, IL-17) producing a context which promotes the formation and maintenance of granulomas. Innate and adaptive immunity are both involved in the immune response in sarcoidosis. Th1 cells, producing IFN-γ, are crucial in the early stages of granuloma formation, while Th17 cells are involved in chronic inflammation and fibrosis of advanced sarcoidosis [17]. So, the gold standard for diagnosis of sarcoidosis, according to current guidelines, consists of a combination of clinical, radiological, and histopathological elements. The diagnosis is usually formulated based on a compatible clinical and radiological presentation, histological demonstration of non-caseating granulomas, and the exclusion of other causes of granulomas. Therefore, the diagnosis of sarcoidosis is complex, and in some cases, obtaining histological confirmation is challenging, and patient assessment can be difficult, particularly since the pathophysiology of this disease is not fully understood. The study of microcirculation can thus serve as a diagnostic tool that complements the diagnosis and helps to understand better the patient, not only in terms of diagnostics but also in terms of therapeutic response, as already happens in systemic sclerosis.

### 1.5. Blood Biomarkers for Lung Fibrosis in Sarcoidosis

Lung fibrosis, particularly idiopathic pulmonary fibrosis (IPF), and sarcoidosis are two distinct interstitial lung diseases characterized by varying degrees of lung inflammation and scarring. Early diagnosis and monitoring of disease progression are crucial for treatment efficacy and improving patient outcomes. Recent advancements in the identification of blood biomarkers have shown promise in providing insights into the pathophysiological processes of these conditions. New biomarkers which could predict disease progression and complications like lung fibrosis have been identified helping in understanding the disease’s pathogenesis [15,16,17,18,19]. Here is a summary of some potential biomarkers for each condition, in particular, regarding lung fibrosis:

a- Signal Peptide-Cubilin mRNA (SP-C): Elevated levels have been associated with idiopathic pulmonary fibrosis (IPF) and could serve as a diagnostic marker.
b- Krebs Von Den Lungen-6 (KL-6): A mucin-like glycoprotein, KL-6 levels are often elevated in patients with interstitial lung diseases, particularly IPF, and correlate with disease activity [18,19].
c- Surfactant Proteins (SP-A and SP-D): These proteins are involved in lung surfactant homeostasis; their elevated serum levels can indicate lung injury and are linked with fibrotic diseases [18,19].
d- Matrix Metalloproteinases (MMPs): Specifically, MMP-7 has been identified as a potential biomarker in pulmonary fibrosis [18,19].
e- Bone Morphogenetic Protein (BMP) plasma levels: Changes in BMP levels may correlate with fibrotic processes [18,19].
f- Circulating fibrocytes: These progenitor cells are involved in tissue repair and fi-brosis, detectable in peripheral blood [18,19].

Furthermore, in sarcoidosis, several other biomarkers are identified, as: follows:

a- Angiotensin-Converting Enzyme (ACE): Elevated serum levels of ACE are com-monly observed in sarcoidosis and have been used as a marker for disease activity.
b- Soluble Interleukin-2 Receptor (sIL-2R): High levels in serum can indicate T-cell activation and may correlate with disease activity and severity.
c- C-reactive Protein (CRP): Though not specific, CRP can indicate systemic inflammation and might support diagnosis when elevated.
d- Vascular Endothelial Growth Factor (VEGF): Elevated levels have been associated with sarcoidosis and can reflect disease activity.
e- Interferon gamma (IFN-γ): Enhanced levels may play a role in the granulomatous response seen in sarcoidosis patients and can serve as an indirect biomarker.

In conclusion, these biomarkers show promise for diagnosing and monitoring lung fibrosis and sarcoidosis; however, further research studies are needed to validate their clinical utility and develop standardized testing protocols. Accurate and reliable biomarkers can greatly enhance patient management by helping to guide treatment decisions and assess disease progression [15,16,17,18,19].

## 2. Sarcoidosis and Capillaroscopy

We searched for the key terms “sarcoidosis” and “video-capillaroscopy” in a computerized search of Pub-Med, extending the search back in time without setting limits. We provided a comprehensive overview of the literature, based on a precise evaluation. After our critical analysis, we examined the six yielded works looking for answers to our questions. Finally, we focused on articles about the use of NVC in sarcoidosis comparing methods and results. The oldest work, “Coexistence of systemic sclerosis and sarcoidosis”, dated 2016, is a case report about overlap syndrome of SSc with SA. A skin biopsy was taken and the noncaseating granulomatous structure of the skin was consistent with granulomatous dermatitis, sarcoidal type. Based on the clinical, laboratory, and histological outcomes, the patient was diagnosed with sarcoidosis and SS [41]. Also, the case-report of Alkutobi et al. in 2020 described histological evidence of sarcoidosis in the presence of abnormal nailfold capillaroscopy and negative autoimmune profile [42].

Snow et al. reported results from a survey on capillaroscopy conducted among Scleroderma Clinical Trials Consortium members in 2019 in the US. NVC was performed by most academic practicing SSc specialists (91% of experts), but NVC—the gold standard technique—was rarely performed (only 7%), 64% using a dermatoscope or ophthalmoscope for evaluation, highlighting moreover the need for more formal instruction for NVC. The access to NVC was mainly restricted to centers with special interests, because of the expense and the required expertise when assessing NVC visually [43].

**Figure 5:**
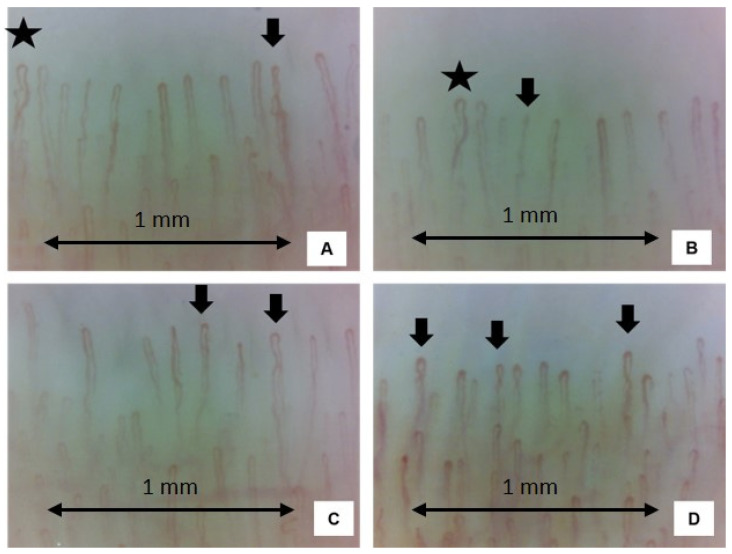
Examples of four nailfold video-capillaroscopy images recorded among four different sarcoidosis patients, all in the remission stage from five years. (Magnification 200×). Black stars (**A**,**B**) indicate the presence crossing capillaries. Black arrows (**A**–**D**) indicate the tortuosity of capillaries. The capillary count per linear mm results in 9–10 capillaries/mm (normal number) (Operators: B.R. and L.M., Pulmonology Unit, University of Trieste).

In their study Radic and coll. compared expert diagnoses formulated using two different imaging techniques on the same 100 patients with Raynaud’s phenomenon, the dermatoscope—which is more affordable and available—proved to be a good screening method. Compared to the gold standard, which is the capillaroscope, it is equally specific (negative when healthy) but less sensitive (positive when diseased), and capable of discriminating between healthy patients and those who need further investigations [44]. No reference to the use of capillaroscopy in sarcoidosis was explicitly mentioned in these two works. Finally, two studies effectively investigated the use of video-capillaroscopy in sarcoidosis. In Table 2 the main characteristics of the two articles about NVC in sarcoidosis are listed (Figure 5). Plus, in Table 2 and Table 3, some additional findings concerning the two articles are described.

Cattelan et al. conducted a cross-sectional retrospective study comparing NVC findings of 26 patients with sarcoidosis to 30 RPF and 30 healthy age- and sex-matched controls; they highlighted that those patients with sarcoidosis had a higher prevalence of abnormal nailfold capillaroscopy findings compared to healthy and Raynaud’s phenomenon controls, but no specific pattern was recognized. Secondarily, they investigated potential links between NVC findings, with the occurrence of autoantibodies and clinical phenotype of the disease; the mean capillary absolute number negatively correlated with the C-reactive protein concentrations and was positively associated with the forced vital capacity percentage (FVC%). Instead, a negative correlation was detected between serum ACE levels and the presence of capillary dilations. They also spotted different ANA positive sarcoidosis patients, hypothesizing it might suggest autoimmune implications in the disease or the production of autoantibodies reactive to tissue damage [45]. NVC parameters did not show a significant change also when patients were stratified according to previous treatment; the study included both currently and previously treated patients with systemic glucocorticoids at the time of NVC, as also patients previously treated with disease modifying anti-rheumatic drugs (DMARDS) and never treated patients [45].

Acemoğlu and coll. conducted a cross-sectional case–control study comparing NVC findings of 42 sarcoidosis patients and 42 age- and sex-matched patients with SSc and healthy individuals which confirmed the abnormal capillaroscopy findings in sarcoidosis and highlighted the correlation of different findings with lung involvement [46]. 

## 3. Discussion

Both articles investigated microvascular alterations in sarcoidosis using the updated criteria to select ill patients: the ATS guidelines for sarcoidosis and ACR/EULAR for systemic sclerosis; both also adopted the gold standard NVC technique ensuring the best sensitivity and specificity ratio, as highlighted in the study of Radic and coll [44]. They also followed the standardized NVC protocol; as an essential tool for rheumatologists, NVC is a patient and operator-dependent technique. Therefore, it requires adherence to a preparation protocol and institutional/official training to ensure maximum efficiency [26].

In the first study, sarcoidosis was compared to two other non-scleroderma patterns of Raynaud’s phenomenon and healthy patients. In the second study, sarcoidosis was compared to both healthy patients and the scleroderma pattern. In both studies the compared cohorts were age- and sex-matched [45,46].

The numerosity of the cohort in the work of Acemoglu was higher, but the results about microvascular damages are like the study of Cattelan. Indeed, both studies found a greater number of microvascular alterations in scleroderma patients compared to healthy controls, supporting the previous hypothesis of microvascular damage in sarcoidosis [19,21], where in fact the activation of the angiogenic pathway and an imbalance between angiogenesis and angiostasis involving VEGF have already been described [47,48,49].

**Figure 6:**
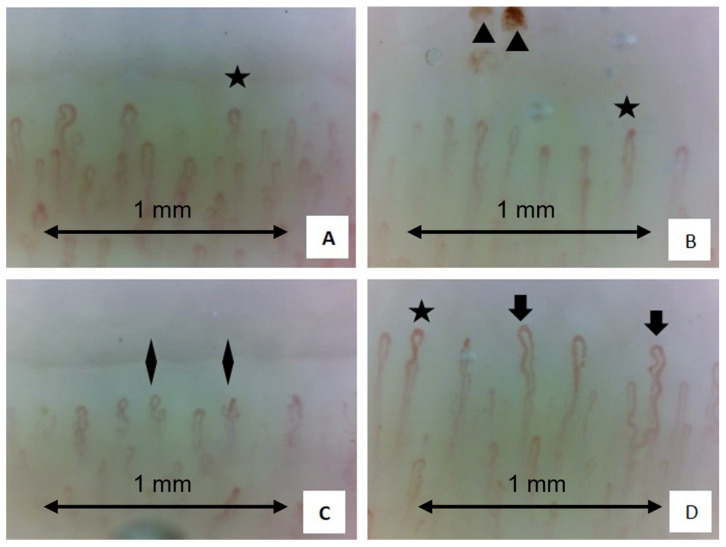
Examples of four nailfold video-capillaroscopy images recorded among four different sarcoidosis patients (**A**–**D**) all in the active stage of disease. (Magnification 200×). Black stars (**A**–**C**) indicate the presence crossing capillaries. Black arrows (**D**) indicate the tortuosity of capillaries. Black triangles (**B**) show the presence of hemosiderin deposits due to microhemorrhages. Black rhombus (**C**) indicates the presence of “bushy” capillaries. The capillary count per linear mm results in 7 capillaries/mm (slightly reduced) (Operators: B.R. and L. M., Pulmonology Unit, University of Trieste).

Currently, the results of both studies supported the angiogenic imbalance as a pathophysiological factor of the disease, which occurred in response to the reduction in the number of capillaries (Figure 6).

Specifically, for both studies, the video-capillaroscopic findings in sarcoidosis were found to be non-scleroderma-like [25].

Furthermore, Cattelan’s study highlighted that these alterations are greater in sarcoidosis compared to patients with Raynaud’s phenomenon (RP), with a higher trend of microhemorrhages and sparser microcapillaries, despite not describing a specific pattern [45]. Acemoglu et al., however, found particularly increased rates in tortuosity and crossing capillaries, suggesting those as a potential additional element in the diagnosis of sarcoidosis [46].

Moreover, in the first study, a significant positive correlation was identified between the absolute capillary count on average and the FVC%, while in the second study, the FVC% was correlated with the increase in crossing capillaries, allowing the hypothesis that microvascular alterations in VCS indicate pulmonary involvement, with airway obstruction having become a known feature of sarcoidosis in recent years [50,51]. Other studies, mostly focused on SSc patients with interstitial lung disease (ILD), highlighting the usefulness of NVC in early detection of lung involvement in connective tissue diseases (CTD) by finding lower capillary density and more bushed/ramified capillaries, as well as giant capillaries which might indicate reduced lung capacity for carbon monoxide [52,53,54,55,56,57].

Corrado A et al. compared NVC findings among patients with idiopathic ILD, SSc-ILD, and chronic obstructive pulmonary disease (COPD). They observed more significant capillary density reduction and neo-angiogenetic aspects in SSc-ILD than idiopathic ILD patients corroborating the potential prognostic role of NVC in diagnosing CTD with lung involvement as the onset manifestation [58].

## 4. Conclusions

Basing on highlighted findings, NVC appears to be a useful tool in the initial evaluation of sarcoidosis patients. Changes in the nailfold capillaries, could indicate disease progression and lung involvement. However, capillaroscopy is useful in the evaluation of the coexistence of sarcoidosis and scleroderma spectrum disorder or overlap syndromes.

To date, no specific pattern has been described for sarcoidosis, and further research is needed to fully understand the implications of nailfold capillaroscopy findings in this disease and to establish standardized guidelines for its use in clinical practice.

## Figures and Tables

**Table 1:** Normal caratheristic of nailfold bed.

Capillary Caratheristics	Normal Findings
**Morphology**	Hairpin or U-shape, with some degree of tortuosity frequent in healthy subjects
**Density (nr capillary/mm)**	9–13/mm (average 9/mm)
**Limb dimensions**	6–19 μm (average 11 μm) for afferent limbs8–20 μm (average 12 μm) for efferent limbs
**Venous limb to arterial limb diameter ratio**	>2:1
**Subpapillary venular plexus (SPVP)**	Visible, in approximately one third of subjects
**Capillary loops**	<35 μm
**Microhemorrhages**	Single hemosiderin store in normal capillaries can be observed in healthy subjects, especially after trauma

**Table 2:** Selected articles.

Article	Study Methods	Exclusion Criteria	Capillaroscopic Strategy and Methods	Capillaroscopy Findings
**Microvascular capillaroscopic abnormalities and occurrence of antinuclear autoantibodies in patients with sarcoidosis.** **[45]**	Files from an NVC database were extracted for 26 histologically diagnosed sarcoidosis and 30 PRP patients, age- and sex-matched. 30 HCs mainly recruited among health professionals willing to participate to the study. NVC figures, detailed analysis and scores, baseline performance, complete medical history and laboratory findings of all enrolled patients were achieved from patient file.	<18 years old, active smokers, underlying malignancies, systemic untreated infections (i.e., HBV, HCV and HIV), overlapping CTD, diabetes, severe uncontrolled systemic hypertension and peripheral atherosclerotic diseases. Other overlapping autoimmune diseases which could have biased the results of the immunological profile (ANA and ENA).	NVC was performed using a 200× magnification optical probe connected to an image analysis software (DS Medica Srl Videocap ©, Ver 10.00.13, Milan, Italy), evaluation by the same physician, blinded to the patient’s clinical history. Waited for a minimum of 15 min in a room at a temperature range of 20–22 °C before NVC. Two digital pictures of two-millimetre area in the middle of the nailfold bed of eight fingers, thumbs excluded, collected for each subject. Capillary density calculated with the same standardized methodology, considering all the 16 images collected for each subject.	Giant capillaries were not reported in any group. SA patients displayed a significantly higher rate of capillary dilations than HC (*p* for trend = 0.046). Mean lower capillary number for mm in comparison with both PRP and HC (*p* < 0.001). SA had higher percentage of abnormal shapes than both PRP patients (*p* < 0.001) and HC (*p* = 0.003). Microhemorrhages frequency showed no statistically significant difference through a direct comparison of the three subgroups, but a trend of increase in the percentage of microhemorrhages in SA patients > PRP patient > HC NVC parameters did not significantly change when stratifying patients according to previous treatment.
**Microvascular damage evaluation based on nailfold video-capillaroscopy in sarcoidosis.** **[46]**	42 biopsy proven sarcoidosis patients and 42 age- and sex-matched patients with SSc and healthy individuals underwent NVC	Interstitial lung disease other than sarcoidosis and systemic sclerosis, primary Raynaud’s phenomenon, obesity (BMI ≥ 30), diabetes mellitus, heart failure, coronary artery disease, pulmonary hypertension, oxygen saturation < 92 on room air, pregnancy, malignancy, haematological diseases.	NVC with digital microscope (Dino-Lite CapillaryScope 200, MEDL4N PRO, Almere, Netherlands) and software program (DinoCapture v2.0 software from AnMo Electronics Corp.) The same, blinded to the study groups certified investigator. Not smoked in the last half hour set down in a room with a temperature of 22–25 C° for at least 15 min before NVC. ×200 magnification NVC technique. The second-Fifth fingers of both hands were evaluated.	Median capillary density similar in SA and HC groups, lower in patients with SSc compared to other groups (*p* < 0.001). In patients with SA and elongated capillary ratio, rate of tortuosity and crossing capillaries of 50% and above statistically significantly higher than patients with SSc and HC. Hemorrhage, dilated capillary, avascular area, and neoangiogenesis higher in patients with SSc compared to other groups (*p* < 0.05).

Legend. PRP: primary Raynaud’s phenomenon, HC: healthy control.

**Table 3:** Additional findings.

Articles	Autoantibodies	PFT	Laboratory Investigations	Correlating NVC Variables with Other Features
**Microvascular capillaroscopic abnormalities and occurrence of antinuclear autoantibodies in patients with sarcoidosis.** **[45]**	Blood tests for ANA and ENA. PRP patients were, for definition, all negative for ANA and ENA tests. In the prevalence of ANA positivity in SA patients were significantly higher in comparison with PRP patients (*p* = 0.001) and HCs (*p* = 0.015).	SA patients underwent PFTs (including plethysmography) to measure percent predicted forced vital capacity (FVC%), forced expiratory volume in 1 s (FEV1%), diffusing capacity of carbon monoxide (DLCO%) and total lung capacity (TLC%).	(WBC), (Hb), (PLT) (CRP), (25OH-D), (Ca) and (ACE) concentrations, the latter two being considered most correlating with sarcoidosis disease activity. Blood samples collected, at most, 3 months before NVC examination.	Negative correlation between capillary dilations and serum ACE concentrations and between the mean capillary number and CRP serum concentrations, positive correlation between mean absolute capillary count and the FVC%.
**Microvascular damage evaluation based on nailfold video-capillaroscopy in sarcoidosis.** **[46]**	All patients with a diagnosis of sarcoidosis included in the study had negative ANA test results.	FEV1, forced vital capacity (FVC), and diffusion capacity for carbon monoxide (DLCO)] were measured at admission.	The acute phase reactants (ESR and CRP), antinuclear antibody (ANA), hematological (white blood cells, haemoglobin, and thrombocyte), and biochemical test results (creatinine, calcium, albumin, glucose, and uric acid) at the last visit of sarcoidosis patients were obtained from the hospital database.	No significant correlation was found between the laboratory results, CT stage, treatments, disease duration (chronic-acute disease), and capillaroscopy findings of the patients with SA. In patients with SA, the FEV1 value was lower in patients with a crossing rate > 50% than in those with a crossing ratio below 50%.

Legend. (PRP) primary Raynaud’s phenomenon.

## References

[1] Cutolo M., Sulli A., Smith V. (2013). How to perform and interpret capillaroscopy. Best Pract. Res. Clin. Rheumatol..

[2] Cutolo M., Trombetta A.C., Melsens K., Pizzorni C., Sulli A., Ruaro B., Paolino S., Deschepper E., Smith V. (2018). Automated assessment of absolute nailfold capillary number on videocapillaroscopic images: Proof of principle and validation in systemic sclerosis. Microcirculation.

[3] Smith V., Pizzorni C., Riccieri V., Decuman S., Brusselle G., DE Pauw M., Deschepper E., Piette Y., Ruaro B., Sulli A. (2016). Stabilization of Microcirculation in Patients with Early Systemic Sclerosis with Diffuse Skin Involvement following Rituximab Treatment: An Open-label Study. J. Rheumatol..

[4] Crouser E.D., Maier L.A., Wilson K.C., Bonham C.A., Morgenthau A.S., Patterson K.C., Abston E., Bernstein R.C., Blankstein R., Chen E.S. (2020). Diagnosis and Detection of Sarcoidosis. An Official American Thoracic Society Clinical Practice Guideline. Am. J. Respir. Crit. Care Med..

[5] Drent M., Crouser E.D., Grunewald J. (2021). Challenges of Sarcoidosis and Its Management. N. Engl. J. Med..

[6] Arkema E.V., Cozier Y.C. (2020). Sarcoidosis epidemiology: Recent estimates of incidence, prevalence and risk factors. Curr. Opin. Pulm. Med..

[7] Ungprasert P., Crowson C.S., Matteson E.L. (2017). Influence of Gender on Epidemiology and Clinical Manifestations of Sarcoidosis: A Population-Based Retrospective Cohort Study 1976–2013. Lung.

[8] Birnbaum A.D., Rifkin L.M. (2014). Sarcoidosis: Sex-Dependent Variations in Presentation and Management. J. Ophthalmol..

[9] Judson M.A. (2015). The Clinical Features of Sarcoidosis: A Comprehensive Review. Clin. Rev. Allergy Immunol..

[10] Baughman R.P., Teirstein A.S., Judson M.A., Rossman M.D., Yeager H., Bresnitz E.A., DePalo L., Hunninghake G., Iannuzzi M.C., Johns C.J. (2001). Clinical Characteristics of Patients in a Case Control Study of Sarcoidosis. Am. J. Respir. Crit. Care Med..

[11] Torregiani C., Reale M., Confalonieri M., Dore F., Crisafulli C., Baratella E., Salton F., Confalonieri P., Ruaro B., Maiello G. (2024). Cardiopulmonary exercise testing complements both spirometry and nuclear imaging for assessing sarcoidosis stage and for monitoring disease activity. Sarcoidosis Vasc Diffuse Lung Dis..

[12] Chevalet P., Clément R., Rodat O., Moreau A., Brisseau J.-M., Clarke J.-P. (2004). Sarcoidosis Diagnosed in Elderly Subjects: Retrospective study of 30 cases. Chest.

[13] Ungprasert P., Carmona E.M., Utz J.P., Ryu J.H., Crowson C.S., Matteson E.L. (2015). Epidemiology of Sarcoidosis 1946–2013: A Population-Based Study. Mayo Clin. Proc..

[14] Rizzato G., Palmieri G., Agrati A.M., Zanussi C. (2004). The organ-specific extrapulmonary presentation of sarcoidosis: A frequent occurrence but a challenge to an early diagnosis. A 3-year-long prospective observational study. Sarcoidosis Vasc. Diffus. Lung Dis..

[15] Cifaldi R., Salton F., Confalonieri P., Trotta L., Barbieri M., Ruggero L., Valeri G., Pozzan R., Della Porta R., Kodric M. (2023). Pulmonary Sarcoidosis and Immune Dysregulation: A Pilot Study on Possible Correlation. Diagnostics.

[16] Grutters J.C. (2023). Establishing a Diagnosis of Pulmonary Sarcoidosis. J. Clin. Med..

[17] Cameli P., Biondini D., Carleo A., Stock C.J.W. (2023). Editorial: New insights in sarcoidosis: From bench to bedside. Front. Med..

[18] Casanova N.G., Reyes-Hernon V., Gregory T., Sun B., Bermudez T., Hufford M.K., Oita R.C., Camp S.M., Hernandez-Molina G., Serrano J.R. (2022). Biochemical and genomic identification of novel biomarkers in progressive sarcoidosis: HBEGF, eNAMPT, and ANG-2. Front. Med..

[19] Confalonieri P., Volpe M.C., Jacob J., Maiocchi S., Salton F., Ruaro B., Confalonieri M., Braga L. (2022). Regeneration or Repair? The Role of Alveolar Epithelial Cells in the Pathogenesis of Idiopathic Pulmonary Fibrosis (IPF). Cells.

[20] Tuleta I., Skowasch D., Biener L., Pizarro C., Schueler R., Nickenig G., Schahab N., Schaefer C., Pingel S. (2017). Impaired vascular function in sarcoidosis patients. Adv. Exp. Med. Biol..

[21] Aciksari G., Kavas M., Atici A., Kul S., Erman H., Yilmaz Y., Demircioglu K., Yalcinkaya E., Kanbay A., Caliskan M. (2018). Endocan Levels and Endothelial Dysfunction in Patients with Sarcoidosis. Angiology.

[22] Van den Hoogen F., Khanna D., Fransen J., Johnson S.R., Baron M., Tyndall A., Matucci-Cerinic M., Naden R.P., Medsger T.A., Carreira P.E. (2013). 2013 classification criteria for systemic sclerosis: An American college of rheumatology/European league against rheumatism collaborative initiative. Ann. Rheum. Dis..

[23] Ruaro B., Sulli A., Smith V., Pizzorni C., Paolino S., Alessandri E., Trombetta A.C., Cutolo M. (2018). Advances in nailfold capillaroscopic analysis in systemic sclerosis. J. Scleroderma Relat. Disord..

[24] Ingegnoli F., Smith V., Sulli A., Cutolo M. (2018). Capillaroscopy in Routine Diagnostics: Potentials and Limitations. Curr. Rheumatol. Rev..

[25] Smith V., Vanhaecke A., Herrick A.L., Distler O., Guerra M.G., Denton C.P., Deschepper E., Foeldvari I., Gutierrez M., Hachulla E. (2019). Fast track algorithm: How to diferentiate a “scleroderma pattern” from a “non scleroderma pattern”. Autoimmun. Rev..

[26] Sulli A., Ruaro B., Smith V., Pizzorni C., Zampogna G., Gallo M., Cutolo M. (2013). Progression of Nailfold Microvascular Damage and Antinuclear Antibody Pattern in Systemic Sclerosis. J. Rheumatol..

[27] Ma Z., Mulder D.J., Gniadecki R., Tervaert J.W.C., Osman M. (2023). Methods of Assessing Nailfold Capillaroscopy Compared to Video Capillaroscopy in Patients with Systemic Sclerosis—A Critical Review of the Literature. Diagnostics.

[28] Grassi W., Del Medico P., Izzo F., Cervini C. (2001). Microvascular involvement in systemic sclerosis: Capillaroscopic findings. Semin. Arthritis Rheum..

[29] De Angelis R., Cutolo M., Salaffi F., Restrepo J.P., Grassi W. (2009). Quantitative and qualitative assessment of one rheumatology trainee’s experience with a self-teaching programme in videocapillaroscopy. Clin. Exp. Rheumatol..

[30] Herrick A.L., Murray A. (2018). The role of capillaroscopy and thermography in the assessment and management of Raynaud’s phenomenon. Autoimmun. Rev..

[31] Blockmans D., Vermylen J., Bobbaers H. (1993). Nailfold Capillaroscopy in Connective Tissue Disorders and in Raynaud’s Phenomenon. Acta Clin. Belg..

[32] Cutolo M., Sulli A., Secchi M.E., Olivieri M., Pizzorni C. (2007). The contribution of capillaroscopy to the differential diagnosis of connective autoimmune diseases. Best Pr. Res. Clin. Rheumatol..

[33] Maricq H.R., Maize J.C. (1982). Nailfold Capillary Abnormalities. Clin. Rheum. Dis..

[34] Andrade L.E.C., Gabriel A., Assad R.L., Ferrari A.J.L., Atra E. (1990). Panoramic nailfold capillaroscopy: A new reading method and normal range. Semin. Arthritis Rheum..

[35] Zhang R., Li X., Balasundaram G., Li B., Qi Y., Santosa A., Tan T.C., Olivo M., Bi R. (2024). Hybrid Photoacoustic Ultrasound Imaging System for Cold-Induced Vasoconstriction and Vasodilation Monitoring. IEEE Trans. Biomed. Eng..

[36] Kabasakal Y., Elvins D.M., Ring E.F., McHugh N.J. (1996). Quantitative nailfold capillaroscopy findings in a population with connective tissue disease and in normal healthy controls. Ann. Rheum. Dis..

[37] Cutolo M., Matucci Cerinic M. (2007). Nailfold capillaroscopy and classification criteria for systemic sclerosis. Clin. Exp. Rheumatol..

[38] Bernero E., Sulli A., Ferrari G., Ravera F., Pizzorni C., Ruaro B., Zampogna G., Alessandri E., Cutolo M. (2013). Prospective capillaroscopy-based study on transition from primary to secondary Raynaud’s phenomenon: Preliminary results. Reumatismo.

[39] Carpentier P.H., Maricq H.R. (1990). Microvasculature in Systemic Sclerosis. Rheum. Dis. Clin. N. Am..

[40] Maricq H.R., Weinberger A.B., LeRoy E.C. (1982). Early detection of scleroderma-spectrum disorders by in vivo capillary microscopy: A prospective study of patients with Raynaud’s phenomenon. J. Rheumatol..

[41] Godziszewska S., Widuchowska M., Kopeć-Mędrek M., Kucharz E.J. (2016). Coexistence of systemic sclerosis and sarcoidosis. Wiad. Lek..

[42] Alkutobi Z., Sidhu A., Nandagudi A. (2020). O10 A case of sarcoidosis mimicking Sjögren’s syndrome along with abnormal nailfold capillaroscopy. Rheumatol. Adv. Pr..

[43] Snow M.H., Saketkoo L.A., Frech T.M., Stever J.R., Lebedoff N., Herrick A.L., Cutolo M., Smith V. (2019). Results from an American pilot survey among Scleroderma Clinical Trials Consortium members on capillaroscopy use and how to best implement nailfold capillaroscopy training. Clin. Exp. Rheumatol..

[44] Radic M., Snow M., Frech T.M., Saketkoo L.A., Cutolo M., Smith V. (2020). Consensus-based evaluation of dermatoscopy versus nailfold videocapillaroscopy in Raynaud’s phenomenon linking USA and Europe: A European League against Rheumatism study group on microcirculation in rheumatic diseases project. Clin. Exp. Rheumatol..

[45] Cattelan F., Hysa E., Gotelli E., Pizzorni C., Bica P.F., Grosso M., Barisione E., Paolino S., Carmisciano L., Sulli A. (2022). Microvascular capillaroscopic abnormalities and occurrence of antinuclear autoantibodies in patients with sarcoidosis. Rheumatol. Int..

[46] Acemoğlu Z., Türk I., Aşık M.A., Bircan A., Deniz P.P., Arslan D., Hanta I., Ünal I. (2023). Microvascular damage evaluation based on nailfold videocapillarosopy in sarcoidosis. Clin. Rheumatol..

[47] Piotrowski W.J., Kiszałkiewicz J., Pastuszak-Lewandoska D., Górski P., Antczak A., Migdalska-Sęk M., Górski W., Czarnecka K.H., Domańska D., Nawrot E. (2015). Expression of HIF-1A/VEGF/ING-4 axis in pulmonary sarcoidosis. Adv. Exp. Med. Biol..

[48] Pabst S., Karpushova A., Diaz-Lacava A., Herms S., Walier M., Zimmer S., Cichon S., Nickenig G., Nöthen M.M., Wienker T.F. (2010). VEGF gene haplotypes are associated with sarcoidosis. Chest.

[49] Michalska-Jakubus M., Cutolo M., Smith V., Krasowska D. (2019). Imbalanced serum levels of Ang1, Ang2 and VEGF in systemic sclerosis: Integrated efects on microvascular reactivity. Microvasc. Res..

[50] Laohaburanakit P., Chan A. (2003). Obstructive Sarcoidosis. Clin. Rev. Allergy Immunol..

[51] Rabahoğlu B., Oymak F.S., Ketencioğlu B.B., Tutar N., Gülmez İ., Yılmaz İ. (2021). Frequency of peripheral blood eosinophilia and obstructive airway disease in sarcoidosis. Turk. J. Med. Sci..

[52] Smith V., Riccieri V., Pizzorni C., Decuman S., Deschepper E., Bonroy C., Sulli A., Piette Y., De Keyser F., Cutolo M. (2013). Nailfold capillaroscopy for prediction of novel future severe organ involvement in systemic sclerosis. J. Rheumatol..

[53] Wu W., Jordan S., Becker M.O., Dobrota R., Maurer B., Fretheim H., Ye S., Siegert E., Allanore Y., Hoffmann-Vold A.M. (2018). Prediction of progression of interstitial lung disease in patients with systemic sclerosis: The SPAR model. Ann. Rheum. Dis..

[54] Ruaro B., Confalonieri M., Salton F., Wade B., Baratella E., Geri P., Confalonieri P., Kodric M., Biolo M., Bruni C. (2021). The Relationship between Pulmonary Damage and Peripheral Vascular Manifestations in Systemic Sclerosis Patients. Pharmaceuticals.

[55] Castellví I., Simeón-Aznar C.P., Sarmiento M., Fortuna A., Mayos M., Geli C., Diaz-Torné C., Moya P., De Llobet J.M., Casademont J. (2015). Association between nailfold capillaroscopy findings and pulmonary function tests in patients with systemic sclerosis. J. Rheumatol..

[56] Caetano J., Paula F.S., Amaral M., Oliveira S., Alves J.D. (2019). Nailfold Videocapillaroscopy Changes Are Associated with the Presence and Severity of Systemic Sclerosis-Related Interstitial Lung Disease. J. Clin. Rheumatol..

[57] Guillen-del-Castillo A., Simeòn-Aznar C.P., Callejas-Moraga E.L., Tolosa-Vilella C., Alonso-Vila S., Fonollosa-Pla V. (2018). Quantitative Videocapillaroscopy Correlates with Functional Respiratory Parameter: A Clue for Vasculopathy as a Pathogenic Mechanism for Lung Injury in Systemic Sclerosis. Arthritis Res. Ther..

[58] Corrado A., Carpagnano G.E., Gaudio A., Foschino-Barbaro M.P., Cantatore F.P. (2010). Nailfold capillaroscopic findings in systemic sclerosis related lung fibrosis and in idiopathic lung fibrosis. Jt. Bone Spine.

[59] D’Oria M., Gandin I., Riccardo P., Hughes M., Lepidi S., Salton F., Confalonieri P., Confalonieri M., Tavano S., Ruaro B. (2022). Correlation between Microvascular Damage and Internal Organ Involvement in Scleroderma: Focus on Lung Damage and Endothelial Dysfunction. Diagnostics.

[60] Ruaro B., Smith V., Sulli A., Pizzorni C., Tardito S., Patané M., Paolino S., Cutolo M. (2019). Innovations in the Assessment of Primary and Secondary Raynaud’s Phenomenon. Front. Pharmacol..

[61] Hsu H.-C., Wang L., Wang L.V. (2016). In vivo photoacoustic microscopy of human cuticle microvasculature with single-cell resolution. J. Biomed. Opt..

[62] Baratella E., Bussani R., Zanconati F., Marrocchio C., Fabiola G., Braga L., Maiocchi S., Berlot G., Volpe M.C., Moro E. (2021). Radiological–pathological signatures of patients with COVID-19-related pneumomediastinum: Is there a role for the Sonic hedgehog and Wnt5a pathways?. ERJ Open Res..

[63] Bi R., Zhang R., Meng L., Du Y., Low J., Qi Y., Rajarahm P., Lai A.Y.F., Tan V.S.Y., Ho P. (2024). A portable optical pulsatile flowmetry demonstrates strong clinical relevance for diabetic foot perfusion assessment. APL Bioeng..

[64] Ruaro B., Confalonieri P., Santagiuliana M., Wade B., Baratella E., Kodric M., Berria M., Jaber M., Torregiani C., Bruni C. (2021). Correlation between Potential Risk Factors and Pulmonary Embolism in Sarcoidosis Patients Timely Treated. J. Clin. Med..

[65] Mansueto N., Rotondo C., Corrado A., Cantatore F.P. (2021). Nailfold capillaroscopy: A comprehensive review on common findings and clinical usefulness in non-rheumatic disease. J. Med. Investig..

